# Built environment approaches: Extension personnel's preferences, barriers, and facilitators

**DOI:** 10.3389/fpubh.2022.960949

**Published:** 2022-10-14

**Authors:** Laura E. Balis, Michelle Grocke-Dewey

**Affiliations:** ^1^Louisville Center, Pacific Institute for Research and Evaluation, Louisville, KY, United States; ^2^Department of Health and Human Development, Montana State University, Bozeman, MT, United States

**Keywords:** implementation strategies, contextual inquiry methods, cooperative extension service, physical activity, community setting

## Abstract

**Introduction:**

Interventions that modify the built environment can increase population physical activity levels and prevent chronic disease. The national Cooperative Extension System is poised to implement built environment approaches (i.e., pedestrian/bicycle infrastructure and enhanced access to physical activity spaces), but implementation strategies (i.e., methods or techniques to move research to practice) are needed to improve uptake. Effective implementation strategies address relevant barriers and capitalize on facilitators. The purpose of this study was to understand 1) barriers and facilitators to implementing built environment approaches in two state Extension systems, 2) preferences for built environment approaches, and 3) preferences for implementation strategies.

**Methods:**

A cross-sectional online survey was used to understand Extension personnel's preferences for and barriers and facilitators to built environment approaches through a mixed-methods study design. This work was informed by anthropological inquiry as the overall research philosophy, and by the Health Impact Pyramid, Leeman et al.'s classification of implementation strategies, and the Consolidated Framework for Implementation Research as the theoretical frameworks. The survey was distributed to eligible Extension personnel (*n* = 42) in two states. Quantitative data analysis consisted of numbers/proportions and Friedman tests. Qualitative analysis was completed through a rapid deductive approach to quickly produce actionable results.

**Results:**

Fourteen respondents (33%) completed the survey. Most had not implemented physical activity interventions in their communities or had implemented only individual-level interventions, though were interested in implementing built environment approaches. Benches, playground improvements, and crosswalks were the most desired approaches, while facilitation, assessing community strengths and needs, and technical assistance were desired implementation strategies. The most common barriers were relative priority and available resources; facilitators were external policy and incentives and implementation climate.

**Discussion:**

Extension personnel are receptive to built environment approaches and engaged with community coalitions. Yet, invested parties prefer individual-level interventions, and agents perceive a lack of resources for implementation. Implementation strategies that build capacity in both the Extension system and community coalitions may address these barriers through increasing relative priority and sharing existing resources. This work is a first step toward compiling implementation strategies to address relevant barriers to built environment approaches in community settings.

## Introduction

Physical inactivity is a modifiable risk factor for multiple chronic diseases (including cancer, diabetes, and cardiovascular disease) (1–3). However, only about half of American adults meet the key guidelines for aerobic physical activity, while nearly 80% are not meeting the Physical Activity Guidelines for Americans for both aerobic and muscle-strengthening activity (3, 4). In Montana and Wyoming, 72 and 76% of adults, respectively, do not meet physical activity guidelines (5). Montana and Wyoming are both large, rural states (6.86 and 5.85 people per square mile, respectively), and both have high proportions of rural residents who lack environmental support for physical activity and are less active than their urban peers (6–10). Evidence-based interventions that promote physical activity, especially in rural areas, are necessary.

Interventions that modify the built environment are an evidence-based approach to increasing physical activity levels. Organizations including the American Academy of Pediatrics, American Heart Association, and Institute of Medicine now recommend interventions at the organizational, community, and policy levels (11). The Community Preventive Services Task Force and Centers for Disease Control and Prevention recommends built environment approaches including activity-friendly routes to everyday destinations (i.e., pedestrian/bicycle infrastructure) and enhanced access to places for physical activity (12–14). These interventions 1) have high reach, 2) can result in long-term changes, and 3) help individuals overcome barriers to physical activity through making the healthy choice the easy choice (14). Overall, environment-level interventions have greater population-level impacts—and require less individual effort to receive benefits—than individual-level approaches (15).

Built environment approaches include evidence-based interventions at both the micro and macro level. While both are effective, micro-level interventions can be implemented more rapidly and typically require less money (13). Rather than encouraging individuals to seek out recreation or competitive athletics—or even structured “workouts”—built environment approaches facilitate regular physical activity through active transportation (e.g., a safe cycling route from home to school or work) and easy access through open-access facilities (e.g., a playground for youth with a walking path for adults). Additionally, implementing micro-level interventions can encourage community groups to implement macro-level change—especially in rural communities (13).

With their strong local ties and commitment to improving health (16–18), the national land-grant university Cooperative Extension System (herein: Extension) is one community-based organization poised to implement built environment approaches. Extension is a well-networked, trusted organization with presence in all 50 states (19). Over the past 8 years, Extension has been expanding their focus area from primarily individual-level programming to also incorporating policy, systems and environment-level interventions (18, 20–23). However, translating complex environmental change interventions to practice is challenging, especially in under-resourced community settings where lack of local buy-in may act as a secondary barrier (24–26).

Implementation strategies (i.e., methods or techniques to move research to practice) are recommended to improve the adoption, implementation, and maintenance of evidence-based interventions (27). For example, training and education, changing infrastructure, financial strategies, and iterative assistance are commonly used to increase the uptake of evidence-based interventions (27). Yet, there is a lack of research on implementation strategies for community (vs. clinical) settings, and little is known about effective strategies for the integration of built environment approaches (28–32).

First, for implementation strategies to be effective, they needed to address relevant barriers and capitalize on facilitators. However, the status quo in supporting community settings to implementing built environment approaches is providing top-down or “push” implementation strategies (33) such as funding, training, and technical assistance for prioritized communities—without first assessing implementation determinants and selecting relevant strategies. Implementation strategies that address the wrong level of barriers (e.g., providing training when delivery agents face organizational barriers) fail to be effective (34). Traditionally, funders provide familiar implementation strategies without understanding local contextual factors, that often vary place to place (34). No single implementation strategy—even approaches such as training that are commonly used in community settings—are superior, or even needed, in all situations, yet a “catch-all approach” is typically deferred to (34). Little is known about Extension personnel's barriers and facilitators to built environment approaches. What is known comes from investigations in state Extension systems in the southern region, which have different missions, visions, and values than those in the western region (21, 35–37). Understanding contextual factors for each unique setting is key to selecting relevant implementation strategies, and is considered a necessary first step in implementation research (38–40). Thus, there is a need for a better understanding of Extension personnel's barriers and facilitators to implementing built environment approaches in Montana and Wyoming.

Related, for implementation strategies to succeed, they need to fit the unique needs and resources of their intended settings and be considered feasible and important by the personnel and settings they affect (39). Extension professionals' perceptions of potential implementation strategies were also unknown. Finally, preferences for built environment approaches were also deemed important to informing future implementation strategies. That is, more information about the specific built environment approaches Extension professionals are more likely to adopt (e.g., bike racks vs. midblock crossings) is needed to design and tailor implementation strategies, and these perceptions had not yet been investigated.

Taken together, little is known about Extension personnel's barriers and facilitators to implementing built environment approaches, preferences for implementation strategies, or preferences for the built environment approaches these strategies would support. The purpose of this study was to understand 1) barriers and facilitators to implementing built environment approaches in two state Extension systems, 2) preferences for potential implementation strategies, and 3) preferences for micro-level built environment approaches. A secondary purpose was to assess Extension personnel's interest in joining an integrated research-practice partnership (41, 42) to collaboratively select, tailor, and test implementation strategies. The expected contributions of this manuscript are to lay the foundation for selecting, tailoring, and testing community-driven implementation strategies through a research-practice partnership approach (38, 41).

## Methods

### Study design

A mixed-methods study design was used to understand Extension personnel's preferences for and barriers and facilitators to built environment approaches through a cross-sectional online survey. This study deemed exempt by the Pacific Institute for Research and Evaluation Institutional Review Board.

This work was informed by anthropological inquiry (43, 44) as the overall research philosophy, and by the Health Impact Pyramid (15), Leeman et al.'s classification of implementation strategies (33), and the Consolidated Framework for Implementation Research (CFIR) (45) as the theoretical frameworks. Anthropological free listing and ranking methodology and open-ended questions were used to garner insight from respondents through an emic, or local, perspective (43, 44). This approach facilitated an understanding of contextual factors from the perspective of the participants (agents who could implement the built environment strategies) rather than the observers (researchers) (46).

The Health Impact Pyramid describes the population health impact vs. individual effort needed to benefit at multiple intervention levels (15). It was used to classify potential interventions delivered by Extension agents as individual-level (education; lower population impact) vs. environment level (changing the environment or context to make it safe and easy for people to be active; higher impact) (15). Leeman et al. categorize implementation strategies by identifying actors (who enacts the strategy) and actions (levels and determinants targeted) (33). Within this framework, capacity-building strategies are those that target “motivation and capability to engage in implementation process strategies (in general, not related to a specific EBI)” through influencing individuals and processes (33)—which aligns with the need to support agents in selecting from a menu of options to change the built environment. Implementation process strategies target “how well teams execute activities required to select, adapt, and integrate EBIs” and are also EBI-agnostic (33). Thus, capacity building and implementation process strategies were proactively chosen as the most relevant to support agents in selecting and implementing built environment approaches from a menu of options to meet community needs.

### Participants and recruitment

The research team distributed the survey within Montana State University Extension (MSUE) and University of Wyoming Extension (UWE). County-based Extension personnel (called agents in Montana and educators in Wyoming; herein: agents) who could potentially implement built environment approaches were eligible to complete the survey (*n* = 35 in Montana, *n* = 4 in Wyoming). The survey was distributed through saturation sampling (i.e., all members of the population were invited to participate) (21). In UWE, due to the small number of agents with relevant duties, one survey respondent requested that specialists (faculty with state-level Extension duties) with relevant roles (*n* = 3) also be included in the sample, for a total of 42 surveys distributed across both states. Eligible personnel were sent an email inviting them to participate in the survey, with a follow-up email sent 1 week later. The survey was open for 2 weeks. Compensation for completing the survey was not provided. The recruitment email text encouraged agents to provide input and lead to future collaborative projects.

### Data collection

The survey was designed to query aspects of the implementation context that may facilitate or impede implementation of built environment approaches and provide insight into which types of built environment approaches are desired to implement in local communities. Respondents were asked about 1) their current practices and interest in implementing physical activity interventions (multiple response options), 2) interest in implementing specific micro-level built environment approaches (ranked choice item) (12–14, 47), 3) interest in specific implementation strategies (ranked choice item) (33), and 4) barriers and facilitators to implementing built environment approaches (open-ended). Finally, respondents were asked whether they were interested in participating in a collaborative partnership to adapt and test implementation strategies and were asked to provide contact information if interested. Of note, demographic information was not captured in an effort to reduce survey fatigue and ensure confidentiality.

### Data analysis

Statistical analysis was conducted using SPSS (IBM, version 26, Armonk, NY, 2019). Quantitative data analysis consisted of numbers/proportions for the current practices and interest items, and Friedman tests to obtain the mean rankings for the rank choice items. For qualitative analysis, the open-ended barriers and facilitators questions were analyzed through a rapid deductive approach selected to quickly produce actionable results (48). First, the researchers created coding templates based on CFIR constructs within each of the five domains of the framework: intervention characteristics, outer setting, inner setting, characteristics of individuals, and process (45). Second, the responses to the open-ended survey questions were entered into the template (one response per line). Third, each coder independently coded for the presence of CFIR constructs. Lastly, the researchers reconciled discrepancies and came to agreement on final coding. Audit trails (49) were maintained for all data, including templates and all coding documents.

## Results

### Quantitative

Fourteen respondents (33% of those eligible) completed the survey. Eight were employed within MSUE, two within UWE, and four declined to provide this information. Most reported that they had not implemented physical activity interventions in their communities (*n* = 6, 43%) or had implemented only individual-level interventions (*n* = 5, 36%). Fewer reported having implemented both individual-level and environment-level interventions (*n* = 2, 14%) or only environment-level (*n* = 1, 7%). The majority of respondents were somewhat or extremely interested in implementing more built environment approaches in their communities (*n* = 13, 93%) and implementing more individual-level physical activity programming (*n* = 13, 93%).

Results from the built environment approach ranking question indicate that benches (mean 9.0, SD 3.14), playground improvements (mean 8.0, SD 3.96) and crosswalks or mid-block crossings (mean 7.1, SD 3.48) were the most desired micro-level approaches (see Table 1). Park improvements (mean 5.8, SD 3.11), pedestrian signs (mean 5.1, SD 3.68) and landscaping or beautification projects (mean 5.0, SD 3.42) were ranked as least desired. Survey respondents wrote in additional desired built environment approaches: sidewalks / paved multiuse paths (*n* = 2), path maintenance (*n* = 1), and curb extensions (*n* = 1).

**Table 1:** Ranked order of desired built environment approaches.

**Micro-level built environment approach**	**Mean (±SD)**
Benches (e.g., along a walking/biking path)	9.0 (**±**3.14)
Playground improvements	8.0 (**±**3.96)
Crosswalks or mid-block crossings	7.1 (**±**3.48)
Bike racks	6.9 (**±**3.45)
Shared use agreements (e.g., permission to use school or homeowners' association facilities)	6.9 (**±**4.15)
Painted intersections (to slow traffic and promote walking)	6.7 (**±**2.49)
Lighting along streets or walking/biking paths	6.6 (**±**3.48)
Safe routes to school	6.5 (**±**2.75)
Park improvements	5.8 (**±**3.11)
Pedestrian signs (e.g., wayfinding signs with distance and time to walk or bike to destinations)	5.1 (**±**3.68)
Landscaping or beautification projects (e.g., downtown cleanup, planting trees or flowers, adding art or decorative items)	5.0 (**±**3.42)

Regarding desired implementation strategies, agents were most interested in facilitation (mean 5.9, SD 1.75), assessing community strengths and needs (mean 5.6, SD 2.22), and technical assistance (mean 5.2, SD 2.11). They were least interested in new funding (mean 2.6, SD 1.44) (see Table 2). Lastly, 10 respondents (71%) indicated interest in joining the collaborative partnership to adapt and test implementation strategies and provided their contact information.

**Table 2:** Ranked order of desired implementation strategies.

**Implementation strategies**	**Mean (±SD)**
Facilitation (interactive problem solving and support)	5.9 (±1.75)
Assessing community strengths and needs	5.6 (±2.22)
Technical assistance (support following training)	5.2 (±2.11)
Adapting current built environment approaches to meet local needs	5.1 (±2.25)
Educational materials (e.g., manuals or toolkits)	4.8 (±2.27)
Training	3.7 (±2.05)
Engaging community members	3.2 (±1.67)
New funding (e.g., mini-grants)	2.6 (±1.44)

### Qualitative

Survey participants provided responses to their barriers (*n* = 10, 71%) and facilitators (*n* = 8, 57%) to implementing built environment approaches. Each response was coded as one or more CFIR constructs, yielding a total of 21 unique barriers and 21 unique facilitators. The most common barriers were CFIR constructs of relative priority (*n* = 3), available resources (*n* = 3), cost (*n* = 2), and knowledge and beliefs about the intervention (*n* = 2). The most common facilitators were external policy and incentives (*n* = 6) and implementation climate (*n* = 3).

## Discussion

These results indicate that Extension personnel in Montana and Wyoming primarily have experience with individual-level physical activity interventions, but are interested in integrating built environment approaches into their work. Agents were interested in implementing components of both activity-friendly routes to everyday destinations (benches, crosswalks, and bike racks) and access to places for physical activity (playground improvements and shared use agreements). This is an important finding, considering implementing a combination of these types of interventions is recommended (13, 47). Implementing built environment approaches is complex, and to this end, agents indicated that they experience both barriers and facilitators to engaging in this work.

The most prevalent barriers were relative priority and available resources, while the most prevalent facilitators were external policy and incentives (specifically, the presence of community coalitions) and implementation climate. Taken together, Extension agents are receptive to built environment approaches and most are already engaged with community coalitions. Yet, invested parties prefer individual-level approaches to physical activity promotion, and agents perceive a lack of resources (time and personnel) to implement built environment approaches. Thus, implementation strategies that build capacity in both Extension personnel and community coalitions have the potential to overcome these barriers through increasing relative priority and sharing resources that exist within community coalitions.

The primary implementation strategies preferred by agents (facilitation, assessing community strengths and needs, and technical assistance) may be effective in meeting these goals. Providing guidance on community assessments could increase priority through demonstrating needs (e.g., combining secondary data from County Health Rankings with primary data from key informant interviews to demonstrate both an empirical lack of access to physical activity opportunities and community members' perceptions around access) (50). Needs assessment methods differ by state, and research from other state Extension systems revealed that typical needs assessment processes may be inadequate for capturing needs for environment-level changes, especially in health disparate populations (51).

Technical assistance is commonly used to support the implementation of environment-level physical activity interventions in community settings, including Extension (52–55). However, the definition of technical assistance is broad, as it includes assistance with both the evidence-based intervention and the implementation processes (27, 33). To effectively offer technical assistance and produce generalizable evidence for community settings, we suggest defining whether technical assistance is designed to support uptake of a specific evidence-based intervention or implementation process. In the future, the field may benefit from new terminology defining each type of technical assistance in community-friendly terms (32).

There is less research on the use of facilitation—defined as interactive problem solving and support (27)—to assist Extension practitioners. This may be due to differences in terminology, especially between clinical and community settings (32). It is unknown if facilitation has been used within Extension (perhaps under the umbrella of technical assistance). Overall, there is a need for better operationalizing (56) of specific implementation strategies used in Extension and other community settings.

Finally, while there is little similar research to compare this study to, it is interesting to note the differences in barriers and preferred implementation strategies in other states. In two investigations in Louisiana, relative priority, available resources and knowledge of the intervention were also perceived as barriers to environmental change approaches (both nutrition and physical activity) (35, 36). In a similar study conducted in Kentucky and Tennessee, perceptions of shifting from direct education to environmental approaches was again found as a primary barriers, in addition to complexity of the interventions and organization readiness (37). Finally, in a similar study conducted in Arkansas specific to built environment approaches, agents described challenges with community coalitions and a lack of funding as primary barriers to implementing build environment approaches (21). Subsequently, coalition coaching and mini-grants were developed as relevant implementation strategies through a participatory approach (21). In the current study, Extension personnel described coalitions as a facilitator rather than a barrier—although the coalitions' low demand for built environment approaches was seen as a barrier. Interestingly, although agents in Montana and Wyoming viewed cost as a barrier, they were not interested in mini-grants (ranked last in order of interest), while these were deemed a useful approach in Arkansas. Taken together, the diverse findings of each study underscore the importance of contextual inquiry and an emic perspective to understand implementation barriers and facilitators and select relevant implementation strategies. In the future, the results from the present study and the similar studies in the southern region could lead to a rapid, rigorous approach to understanding contextual factors in additional settings through identifying common and unique barriers across additional states and community-based organizations (57, 58).

One recommended approach to selecting implementation strategies to address identified contextual factors is mapping barriers to implementation strategies, as defined by the Expert Recommendations for Implementing Change (ERIC) project (27) through the CFIR-ERIC matching tool (39, 59). Using this tool, up to seven ERIC implementation strategies are suggested for each CFIR construct deemed a barrier (39, 59). We used this tool to identify implementation strategies relevant for the identified barriers in this study and determine whether our prediction of capacity-building and implementation process strategies was accurate. Resultant strategies were related to scale-up and implementation processes. However, the scale-up strategies were related to infrastructure and fees (e.g., payment schemes and easier billing), and were deemed not relevant in a community setting (*N* = 4). The remaining ERIC implementation strategies (*N* = 6), in order of highest cumulative percentage of endorsement, are detailed in Table 3, along with potential examples specific to built environment approaches.

**Table 3:** Potential implementation strategies for built environment approaches identified through CFIR-ERIC matching tool.

**ERIC strategy**	**Built environment example**
Assess for readiness and identify barriers and facilitators	Conduct pre-implementation surveys or focus groups with community partners.
Conduct local consensus discussions	Include community partners in prioritizing built environment approaches to implement.
Capture and share local knowledge	Ask agents who have initial success with built environment approaches to share with community partners. Use shared documents to maintain a list of responses to implementation barriers.
Build a coalition	Deemed not relevant as coalitions exist and are viewed as a facilitator. A potential strategy could support agents in increasing relative priority and sharing resources within coalitions.
Increase demand	Use marketing strategies to inform the priority populations about built environment approaches so they will request from agents (60, 61).
Conduct local needs assessment	Use results of Extension constituent input processes and conduct additional needs assessments (e.g., secondary data from County Health Rankings and primary data from key informant interviews) (51).

Only one of these strategies, conduct local needs assessment was included in the survey (revised to read “assessing community strengths and needs”). The remaining strategies may also be useful in addressing the identified barriers. For example, increasing demand could be used to inform coalition members and the public about built environment approaches to overcome the barrier of low relative priority. Only one strategy, build a coalition, was less applicable to addressing the identified barriers. Overall, our approach of assessing interest in potential implementation strategies before linking contextual factors to strategies may speed the process of selecting and tailoring relevant strategies. Future work is needed to select and tailor these implementation strategies with Extension professionals (e.g., presenting both the highest-ranked strategies and the potential strategies from the CFIR-ERIC matching tool) and determining which are ranked as both feasible and important (39) to move to pilot testing. It may be possible to integrate the two lists of potential strategies (e.g., through providing technical assistance related to conducting local consensus discussions). Finally, these results underscore the need for a compilation of relevant implementation strategies for community (instead of clinical) settings.

### Limitations

A typical process for identifying implementation strategies includes assessing barriers and facilitators, mapping them to implementation strategies, and then following up with delivery agents/implementers to query the feasibility and importance of the strategies (38, 40). Instead, we developed more rapid approach. Based on previous research, Extension agents experience survey fatigue, and one brief survey was the most likely to be completed. We recognize that there may be a need for implementation strategies that were not included in the survey, and that these may require feedback from staff at additional levels (e.g., Extension administrators).

As well, the sample size was small. While all Extension personnel recruited for the survey are eligible to deliver health promotion programming, for many, it is not their primary role. In MSUE, only seven agents have a primary role of delivering health promotion programming. The other 28 eligible agents are the only agents in their counties, meaning their job duties include all disciplines of Extension programming (e.g., 4-H youth development, agriculture, community development, and family consumer sciences/health promotion). In UWE, the seven agents and specialists recruited to complete the survey serve on the Community Vitality and Health initiative team, a merger of one previous team focused on health promotion programming and another on community development. Thus, we may have captured the perceptions of most of the health promotion-focused personnel as well as those focused on other disciplines (e.g., community development) who could collaborate on built environment work.

## Conclusion

Literature on effective implementation strategies for built environment approaches is limited, and little is known about barriers, facilitators, or perceptions crucial to informing this work. The results described here indicate that barriers and facilitators to built environment approaches differ by region. In Montana and Wyoming, are interested in integrating both activity-friendly routes to everyday destinations (benches, crosswalks, and bike racks) and access to places for physical activity (playground improvements and shared use agreements). Barriers to these efforts include relative priority and available resources, while facilitators include the presence of community coalitions and a supportive implementation climate. Potential implementation strategies to address these barriers include facilitation, assessing community strengths and needs, and technical assistance. This work is a significant first step to begin compiling collaborator-informed, community-appropriate implementation strategies to address relevant barriers to built environment approaches in community settings. Ultimately, increasing the uptake of built environment approaches can improve the proportion of community members who meet physical activity guidelines and decrease the burden of chronic disease.

## Data availability statement

The raw data supporting the conclusions of this article will be made available by the authors, without undue reservation.

## Ethics statement

The studies involving human participants were reviewed and approved by Pacific Institute for Research and Evaluation IRB. Written informed consent for participation was not required for this study in accordance with the National Legislation and the Institutional Requirements.

## Author contributions

LB conceived of the study, contributed to its design, and coordination. MG-D contributed to study design, coordination, and manuscript preparation. All authors contributed to the article and approved the submitted version.

## Conflict of interest

The authors declare that the research was conducted in the absence of any commercial or financial relationships that could be construed as a potential conflict of interest.

## Publisher's note

All claims expressed in this article are solely those of the authors and do not necessarily represent those of their affiliated organizations, or those of the publisher, the editors and the reviewers. Any product that may be evaluated in this article, or claim that may be made by its manufacturer, is not guaranteed or endorsed by the publisher.
