# Supplementary material for: Built environment approaches: Extension personnel's preferences, barriers, and facilitators
Source: Front Public Health. 2022 Oct 14;10:960949. doi: 10.3389/fpubh.2022.960949 (PMC9614838; doi:10.3389/fpubh.2022.960949)
Supplement: Supplementary file 1 [file Data_Sheet_1.docx]

**APPENDIX**

**Built environment** approaches to increase physical activity create or modify environmental characteristics in a community to make physical activity easier or more accessible.

This includes both active transportation (such as biking or walking to work) and leisure-time physical activity, with a goal of making physical activity the default, easy option.

For example, creating routes to everyday places through walking paths, bike lanes, crosswalks, and flashing speed limit signs to slow traffic are examples of built environment approaches.

For more information, check out the [CDC's recommendations](https://www.cdc.gov/physicalactivity/activepeoplehealthynation/strategies-to-increase-physical-activity/index.html) for Activity-Friendly Routes to Everyday Destinations and Access to Places for Physical Activity.

1. **Which type of physical activity programs/interventions have you already implemented in your community?** **(select all that apply)**
   1. Individual-level education (e.g. presentations on the benefits of physical activity)
   2. Individual-level classes (e.g. group exercise classes)
   3. Interventions that change the environment or context to make it safe and easy for people to be active (e.g., organizational policies or physical changes to the environment)
   4. I have not implemented any physical activity interventions
2. **I would be: (extremely interested, somewhat interested, not interested, I am already doing enough of this work)**
   1. Interested in doing more built environment physical activity work in my community
   2. Interested in doing more individual-level physical activity programming (e.g. training and/or exercise classes)
3. **Below are some examples of built environment approaches. Please rank them in order of interest in terms of implementing them in your community to promote physical activity (1=most interested, 12=least interested). There is also space for you to write in two additional approaches and rank them along with the examples.**
   1. Bike racks
   2. Crosswalks or mid-block crossings
   3. Painted intersections (to slow traffic and promote walking)
   4. Landscaping or beautification projects (e.g., downtown cleanup, planting trees or flowers, adding art or decorative items)
   5. Pedestrian signs (e.g., wayfinding signs with distance and time to walk or bike to destinations
   6. Safe routes to school
   7. Lighting along streets or walking/biking paths
   8. Traffic calming measures (e.g., speedbumps, flashing speed limit signs)
   9. Shared use agreements (e.g., permission to use school or home owners’ association facilities)
   10. Park improvements
   11. Playground improvements
   12. Benches (e.g., along a walking/biking path)
   13. Other idea: __________
   14. Other idea: __________
4. **If you haven't tried a built environment approach to encourage more physical activity, why not?  If you have, what are some of the barriers that you faced? (These barriers can be on a personal, organizational, community, state, or federal level):** [open-ended]
5. **If you haven’t implemented a built environment approach yet, what strengths and assets would make it easy for you to do so? If you have, what made it easy? (These facilitators can be on a personal, organizational, community, state, or federal level):** [open-ended]
6. **We are planning on offering strategies to support you in implementing built environment approaches. Please rank the following support strategies in terms of which ones you are most interested in (1=most interested, 8=least interested).**
   1. Training
   2. Technical assistance (support following training)
   3. Educational materials (e.g., manuals or toolkits)
   4. New funding (e.g., mini-grants)
   5. Engaging community members
   6. Adapting current built environment approaches to meet local needs
   7. Assessing community strengths and needs
   8. Facilitation (interactive problem solving and support
7. **We are interested in forming a group of agents and specialists/researchers to collaboratively adapt and test strategies to support Agents in implementing built environment approaches. Are you interested in participating?**
8. [If yes to Q7:] **Please provide your name and email address.** [open-ended]
